# Crystal structures of three 1-[4-(4-bromo­but­oxy)­phen­yl] chalcone derivatives: (*E*)-1-[4-(4-bromo­but­oxy)­phen­yl]-3-phenyl­prop-2-en-1-one, (*E*)-1-[4-(4-bromo­but­oxy)­phen­yl]-3-(4-meth­oxy­phen­yl)prop-2-en-1-one and (*E*)-1-[4-(4-bromo­but­oxy)­phen­yl]-3-(3,4-di­meth­oxy­phen­yl)prop-2-en-1-one

**DOI:** 10.1107/S2056989017010052

**Published:** 2017-07-21

**Authors:** Gunasekaran Maragatham, Sivasamy Selvarani, Perumal Rajakumar, Srinivasakannan Lakshmi

**Affiliations:** a Department of Physics, S.D.N.B. Vaishnav College for Women, Chromepet, Chennai 600 044, India; b Department of Organic Chemistry, University of Madras, Guindy Campus, Chennai 600 025, India

**Keywords:** chalcones, crystal structures, mol­ecular conformation, Claisen–Schmidt condensation reaction, hydrogen bonding, C—Br⋯π contact

## Abstract

The crystal structures of three chalcones with a bromo-substituted but­oxy side chain, *viz*. (*E*)-1-[4-(4-bromo­but­oxy)­phen­yl]-3-phenyl­prop-2-en-1-one, C_19_H_19_BrO_2_, (I), (*E*)-1-[4-(4-bromo­but­oxy)­phen­yl]-3-(4-meth­oxy­phen­yl)prop-2-en-1-one, C_20_H_21_BrO_3_, (II), and (*E*)-1-[4-(4-bromo­but­oxy)­phen­yl]-3-(3,4-di­meth­oxy­phen­yl)prop-2-en-1-one, C_21_H_23_BrO_4_, (III), are reported. In all mol­ecules, the conformation of the keto group with respect to the olefinic bond is *s*-*cis*. Mol­ecules of (I) and (II) are nearly planar, while mol­ecule (III) is not planar. In the crystal of compounds (I) and (II), mol­ecules are linked into chains parallel to the *c* axis by C—H⋯π inter­actions. In the crystal of compound (III), mol­ecules are linked by a pairs of C—H⋯O hydrogen bonds, forming inversion dimers. Weak C—Br⋯π inter­actions are also observed in (III).

## Abstract 2

Mol­ecules (I) and (II) are nearly planar, while mol­ecule (III) is not planar. In compounds (I) and (II), mol­ecules are linked into chain by C—H⋯π inter­actions. In compound (III), mol­ecules are linked by a pair of C—H⋯O hydrogen bonds, forming inversion dimers. Weak C—Br⋯π inter­actions are present in (III).

## Chemical context   

Chalcones are 1,3-diphenyl-2-propene-1-one derivatives, in which two aromatic rings are linked by a three carbon α,β-unsaturated carbonyl system. In these materials, the C=O bond acts as an electron-withdrawing group, and electron-rich substituents in the aromatic rings serve as electron-donating groups, forming a so-called *D*—π⋯*A* type mol­ecule. When the electron-rich groups are located on the 4 and/or 4′ positions, the electron flow follows a Λ-shaped path, and therefore the mol­ecule is called a Λ-shaped mol­ecule (Devia *et al.*, 1999 ▸).

Chalcones are abundant in edible plants and are considered to be precursors of flavonoids and isoflavonoids (Patil *et al.*, 2009 ▸). Alk­oxy­lated chalcones have been synthesized by the Claisen–Schmidt condensation reaction (Ghosh & Das, 2014 ▸) using substituted aceto­phenones and aryl­aldehydes in the presence of ethanol and NaOH (Syam *et al.*, 2012 ▸) , methanol and NaOH (Kumar *et al.*, 2010 ▸), methanol and KOH (Bello *et al.*, 2011 ▸), ethanol and KOH (Shenvi *et al.*, 2013 ▸) and Mg(HSO4)_2_ (Maleraju *et al.*, 2013 ▸) under appropriate conditions. Chalcones possess anti­bacterial (Vibhute *et al.*, 2003 ▸), anti­leishmanial (Nielsen *et al.*, 1998 ▸), anti­microbial (Prasad *et al.*, 2006 ▸), anti­tuberculosis (Sivakumar *et al.*, 2007 ▸), anti­tumor (Kumar *et al.*, 2003 ▸), anti­hyperglycemic (Satyanarayana *et al.*, 2004 ▸) and anti­cancer activities (Sweety *et al.*, 2010 ▸). Meth­oxy chalcones exhibit anti-mitotic activity (Go *et al.*, 2005 ▸) and radical scavenging activity (Yayli *et al.*, 2004 ▸). They play a critical role of meth­oxy­lation in both inhibition of breast cancer resistance protein ABCG2 and cytotoxicity (Valdameri *et al.*, 2012 ▸). 2,4-Dihy­droxy-6-meth­oxy-3,5-dimethyl chalcone has (*in vitro*) anti-tumor activity (Ye *et al.*, 2004 ▸), and 2,4-diall­yloxy-6-meth­oxy chalcone has anti-trypanosoma cruzi activity (Aponte *et al.*, 2008 ▸). In 1-(4-benzimidazol-1-yl-phen­yl)-3-(2,4-dimeth­oxy-phen­yl)-propen-1-one chalcone, the presence of meth­oxy groups at positions 2 and 4 appears to be favourable for anti­malarial activity (Yadav *et al.*, 2012 ▸). Chalcones with meth­oxy, dimeth­oxy or trimeth­oxy substituents on one of the phenyl rings exhibit anti­malarial property (Liu *et al.*, 2001 ▸). Of the chalcones possessing meth­oxy and but­oxy side chains, 2,4-dimeth­oxy-4-but­oxy­chalcone exhibits potent activity against the human malaria parasite (Chen *et al.*, 1997 ▸). 1-(4-But­oxy-2-hy­droxy­phen­yl)-3-(2,5-di­meth­oxy­phen­yl) prop-2-en-1-one chalcone has anti­microbial activity (Barot *et al.*, 2013 ▸).
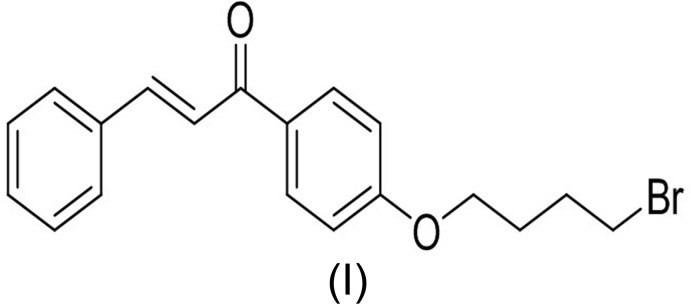
 
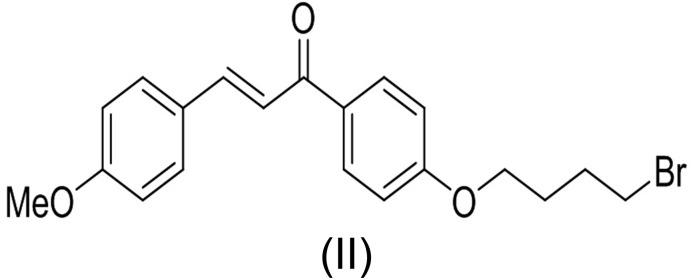
 
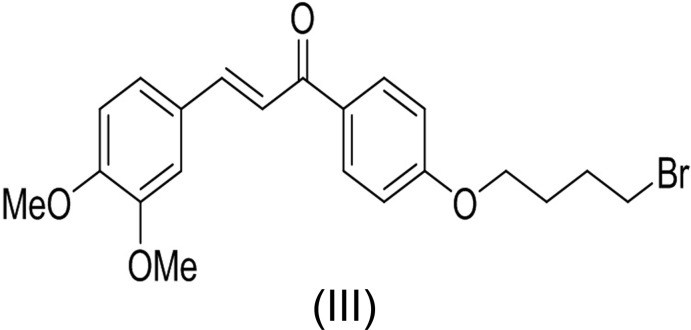


Chalcone compounds are widely used in organic solid photochemistry (Goud *et al.*, 1995 ▸). Chalcone derivatives show non-linear optical (NLO) properties with excellent blue light transmittance and good crystallizability (Shettigar *et al.*, 2006 ▸). The substitution of bromine to *o*-nitro aniline increases its SHG conversion efficiency substanti­ally and is matter of inter­est in research (Bappaliage *et al.*, 2010 ▸). In chalcones, the presence of a bromo substituent is useful to obtain good quality single crystals (Prabhu *et al.*, 2013 ▸). The transparency and the thermal stability of the materials can be improved when the compounds are substituted with a bromo group (Zhao *et al.*, 2000 ▸). Chalcone derivatives with *p*-meth­oxy­phenyl groups possess first order hyperpolarizability and good optical transparency for non-linear optical applications (Muhammad *et al.*, 2016 ▸). In view of the importance of meth­oxy- and bromo-substituted but­oxy side chains in chalcones, the crystal structures of the three title chalcones were determined and analysed.

## Structural commentary   

**Figure 1:**
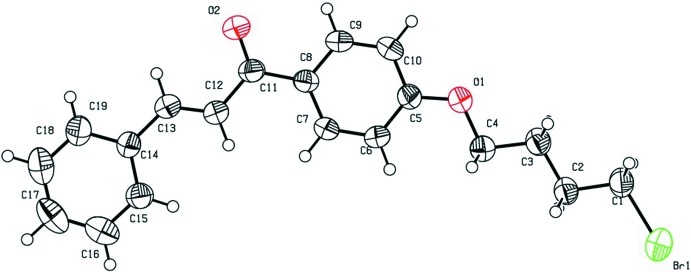
The mol­ecular structure of compound (I), with displacement ellipsoids drawn at the 50% probability level. H atoms are shown as small sphere of arbitrary radius.

The mol­ecular structures of the title compounds (I), (II) and (III) are shown in Figs. 1 ▸, 2 ▸ and 3 ▸, respectively. All three mol­ecules contain a chalcone unit consisting of two phenyl rings (ring *A*: C5–C10; ring *B*: C14–C19) connected by an enone moiety with a bromo­but­oxy side chain attached at the 4-position of one of the phenyl rings. In mol­ecule (I), no other substitution is present, in mol­ecule (II) a meth­oxy side chain is attached to ring B at the 4-position and in mol­ecule (III), two meth­oxy side chains are attached at the 3- and 4-positions of ring *B*. All of them crystallize in the monoclinic space group *P*2_1_/*c* with Z = 4. All three mol­ecules adopt an *s*-*cis* conformation about the central olefinic C12=C13 bond with O2—C11—C12—C13 torsion angles of −3.2 (4), −1.6 (5) and −21.5 (4)°, respectively, and the hydrogen atoms of the central enone groups are *trans*-arranged with respect to the C12=C13 double bond. Mol­ecules (I) and (II) are nearly planar with dihedral angles of 2.32 (13) and 2.33 (15)°, respectively, between the phenyl rings, while mol­ecule (III) is non-planar with a dihedral angle of 50.96 (14)°. The dihedral angles between the atoms of the mean plane of the enone group O2/C11/C12/C13 with rings *A* and *B* are 3.10 (13), 5.34 (11)° in compound (I), 4.45 (13), 5.62 (13)° in compound (II) and 26.70 (11), 24.24 (10)° in compound (III). The increase in these values from compound (I) to compound (III) may be attributed to the presence of meth­oxy substitutents (Chopra *et al.*, 2007 ▸). The meth­oxy groups are twisted slightly from the mean plane of ring *B* with torsion angles of −3.3 (4)° (C20—O3—C17—C16) in mol­ecule (II), 7.3 (4)° (C19—C18—O4—C21) and −9.3 (5)° (C16—C17—O3—C20) in mol­ecule (III).

In compounds (I) and (III), the bromo­alkoxyl tail is roughly co-planar with the attached benzene ring with C6—C5—O1—C4 torsion angles of −0.9 (4) and 2.5 (4)°, respectively. The deviation of the bromo­alkoxyl tail starts from the beginning of the aliphatic chain, as shown by the C5—O1—C4—C3 torsion angles of −179.0 (2) and 177.9 (2)° in (I) and (III), respectively. In compound (II), the bromo­alkoxyl tail is twisted from the attached ring *A* with a C6—C5—O1—C4 torsion angle of 179.7 (3)°.

In compounds (I) and (II), the shortest distances between parallel C=C double bonds are 4.2059 (16) and 4.2881 (18) Å, which are close to the reference value of 4.2 Å for a photo-reactive crystal (Turowska-Tyrk *et al.*, 2003 ▸). In compound (III), the shortest distance between neighbouring ethyl­enic double bonds is 4.6818 (16) Å, indicating that these crystals might be photo inert.

## Supra­molecular features   

**Figure 4:**
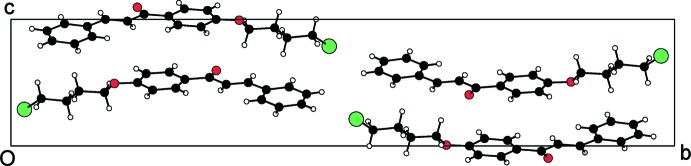
Crystal packing of the compound (I), viewed down the *a* axis.

The packing for mol­ecules (I), (II) and (III) is shown in Figs. 4 ▸, 5 ▸ and 6 ▸, respectively. In the absence of strong hydrogen-bond donors in compounds (I) and (II), the crystal packing is stabilized by weak inter­molecular inter­actions (Nishio *et al.*, 1995 ▸). The involvement of the benzene rings, which are a reservoir of charges in the C—H⋯π inter­action, leads to inter­molecular conjugation (Patil *et al.*, 2013 ▸) and plays an important role in controlling the stereoselectivity of the organic reactions (Nishio *et al.*, 2005 ▸). The C—H⋯π inter­action in compound (I) involves the C2 carbon atom *via* atom H2*A* of ring *A* and the centroid of ring *B* of a symmetry-related mol­ecule (Table 1 ▸), forming chains parallel to the *c* axis. In compound (II), mol­ecules are linked into chains parallel to the *c* axis by two C—H⋯π inter­actions involving the C2 and C3 carbon atoms *via* atoms H2*B* and H3*A* of ring *A* and the centroid of ring *B* of two symmetry-related mol­ecules (Table 2 ▸).

In compound (III), inversion-related mol­ecules are linked into dimers through pairs of inter­molecular hydrogen bonds involving the C10 carbon atom of ring *A via* atom H10 and the O3 oxygen atom (Table 3 ▸). In addition, a non-covalent C—Br⋯*Cg* inter­action involving a lone-electron pair of the Br atom with the anti­bonding orbitals of ring *B* is observed [Br1⋯*Cg* ^ii^ = 3.6577 (12) Å; *Cg* is the centroid of ring *B*; symmetry code: (ii) 1 − *x*, −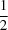 + *y*, 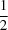 − *z*] having a ‘face-on’ geometry (Imai *et al.*, 2008 ▸). This inter­action plays an important role in generating packing motifs (Prasanna & Guru Row, 2000 ▸; Saraogi *et al.*, 2003 ▸), and it may influence the SHG response of the compound (Harrison *et al.*, 2005 ▸).

## Database survey   

A search of the Cambridge Structural Database (Version 5.36, last update May 2015; Groom *et al.*, 2016 ▸) revealed that the number of compounds based on the chemical unit of chalcone yielded 2168 hits. This involved some compounds with ring closure at the C=C bridge. Avoiding these, the search for the basic unit with two phenyl rings joined by an enone moiety of the title compounds yielded 604 hits. The search for a meth­oxy substitution on one of the phenyl rings of the basic unit gave 124 hits. Extending the search to bromo­meth­oxy, bromo­eth­oxy, bromo­propil­oxy and bromo­but­oxy side chains on the other phenyl ring at the 4- position yielded no hits.

## Synthesis and crystallization   

Chalcone bromides were prepared through condensation of 4-hy­droxy­aceto­phenone (1 equiv.) with benzaldehyde (1 equiv.) for compound (I), 4-meth­oxy­benzaldehyde (1 equiv.) for compound (II) and 4,5-meth­oxy­benzaldehyde (1 equiv.) for compound (III) in 10% NaOH solution (10 ml). After stirring at room temperature for 12 h, the reaction mixtures were poured into ice–water (100 ml), filtered, and the products purified by column chromatography.

Mixtures of chalcone (1 equiv.), 1,4-di­bromo­butane (1.2 equiv.) and anhydrous potassium carbonate (2 equiv.) in dry acetone (40 mL) were then stirred at 333 K for 12 h. After completion of reactions, the solvents were evaporated under reduced pressure and the residues extracted with CH_2_Cl_2_ (3 × 100 ml). The organic layers were separated, washed with brine (1 × 150 ml), dried over anhydrous Na_2_SO_4_ and evaporated to give the crude bromo compounds, which were purified by column chromatography (SiO_2_) using a mixture of hexa­ne/CHCl_3_ (9:2 *v*/*v*) as eluent to afford yellow solids. The compounds were recrystallized by slow evaporation of chloro­form solutions.

## Refinement   

Crystal data, data collection and structure refinement details are summarized in Table 4 ▸. For all compounds, H atoms were localized in difference-Fourier maps and were constrained geometrically with C—H = 0.93, 0.96 and 0.97 Å for aryl, methyl and methyl­ene H atoms, respectively. The *U*_iso_(H) values were set to 1.2*U*_eq_(C) or 1.5*U*_eq_(C) for methyl H atoms.

## Supplementary Material

Crystal structure: contains datablock(s) I, II, III, global. DOI: 10.1107/S2056989017010052/rz5217sup1.cif

Structure factors: contains datablock(s) I. DOI: 10.1107/S2056989017010052/rz5217Isup5.hkl

Structure factors: contains datablock(s) II. DOI: 10.1107/S2056989017010052/rz5217IIsup6.hkl

Structure factors: contains datablock(s) III. DOI: 10.1107/S2056989017010052/rz5217IIIsup7.hkl

Supporting information file. DOI: 10.1107/S2056989017010052/rz5217Isup5.cml

Supporting information file. DOI: 10.1107/S2056989017010052/rz5217IIsup6.cml

Supporting information file. DOI: 10.1107/S2056989017010052/rz5217IIIsup7.cml

CCDC references: 1545201, 1545200, 1545196

Additional supporting information:  crystallographic information; 3D view; checkCIF report

## Figures and Tables

**Figure 2:**
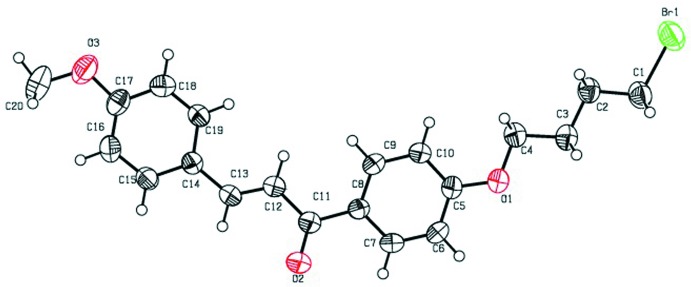
The mol­ecular structure of compound (II), with displacement ellipsoids drawn at the 50% probability level. H atoms are shown as small sphere of arbitrary radius.

**Figure 3:**
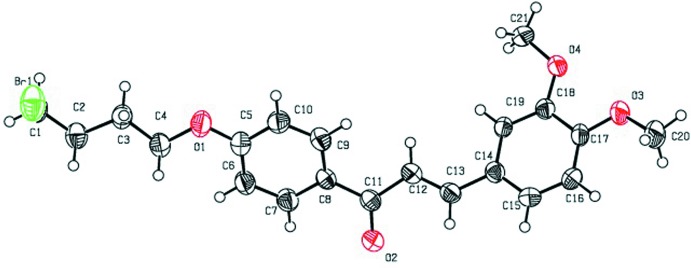
The mol­ecular structure of the compound (III), with displacement ellipsoids drawn at the 50% probability level. H atoms are shown as small sphere of arbitrary radius.

**Figure 5:**
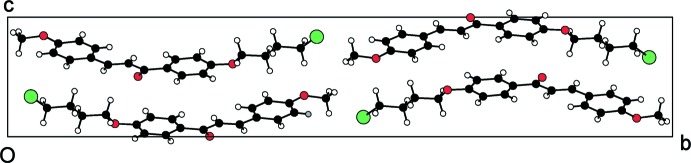
Crystal packing of the compound (II), viewed down the *a* axis.

**Figure 6:**
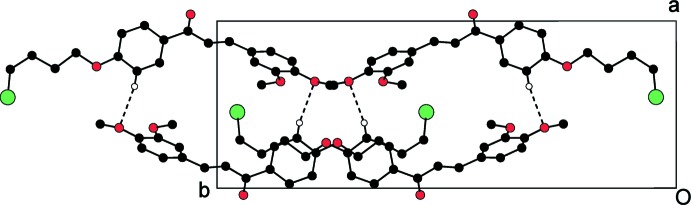
Crystal packing of the compound (III), viewed down the *c* axis. Hydrogen atoms not involved in hydrogen bonding (dashed lines) are omitted.

**Table 1:** Hydrogen-bond geometry (Å, °) for (I) *Cg* is the centroid of the C14–C19 ring

*D*—H⋯*A*	*D*—H	H⋯*A*	*D*⋯*A*	*D*—H⋯*A*
C2—H2*B*⋯*Cg* ^i^	0.97	2.84	3.664 (3)	144

Symmetry code: (i) 
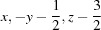
.

**Table 2:** Hydrogen-bond geometry (Å, °) for (II) *Cg* is the centroid of the C14–C19 ring.

*D*—H⋯*A*	*D*—H	H⋯*A*	*D*⋯*A*	*D*—H⋯*A*
C2—H2*B*⋯*Cg* ^i^	0.97	2.87	3.703 (3)	144
C3—H3*A*⋯*Cg* ^ii^	0.97	2.94	3.743 (3)	140

Symmetry codes: (i) 
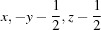
; (ii) 
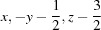
.

**Table 3:** Hydrogen-bond geometry (Å, °) for (III)

*D*—H⋯*A*	*D*—H	H⋯*A*	*D*⋯*A*	*D*—H⋯*A*
C10—H10⋯O3^i^	0.93	2.59	3.505 (3)	169

Symmetry code: (i) 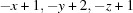.

**Table 4:** Experimental details

	(I)	(II)	(III)
Crystal data			
Chemical formula	C_19_H_19_BrO_2_	C_20_H_21_BrO_3_	C_21_H_23_BrO_4_
*M*_r_	359.25	389.28	419.30
Crystal system, space group	Monoclinic, *P*2_1_/*c*	Monoclinic, *P*2_1_/*c*	Monoclinic, *P*2_1_/*c*
Temperature (K)	296	296	296
*a*, *b*, *c* (Å)	5.8266 (6), 38.743 (4), 7.5613 (7)	5.7331 (3), 41.732 (2), 7.6476 (4)	9.4765 (4), 26.0984 (12), 7.8666 (4)
β (°)	103.257 (3)	101.767 (2)	91.427 (2)
*V* (Å^3^)	1661.4 (3)	1791.28 (16)	1944.98 (16)
*Z*	4	4	4
Radiation type	Mo *K*α	Mo *K*α	Mo *K*α
μ (mm^−1^)	2.48	2.31	2.14
Crystal size (mm)	0.35 × 0.30 × 0.25	0.35 × 0.30 × 0.25	0.35 × 0.30 × 0.25

Data collection			
Diffractometer	Bruker Kappa APEXII CCD	Bruker Kappa APEXII CCD	Bruker Kappa APEXII CCD
Absorption correction	Multi-scan (*SADABS*; Bruker, 2004 ▸)	Multi-scan (*SADABS*; Bruker, 2004 ▸)	Multi-scan (*SADABS*; Bruker, 2004 ▸)
*T*_min_, *T*_max_	0.485, 0.746	0.639, 0.746	0.667, 0.746
No. of measured, independent and observed [*I* > 2σ(*I*)] reflections	23972, 2900, 2144	21484, 3122, 2467	28826, 3434, 2416
*R*_int_	0.039	0.028	0.043
(sin θ/λ)_max_ (Å^−1^)	0.595	0.595	0.595

Refinement			
*R*[*F* ^2^ > 2σ(*F* ^2^)], *wR*(*F* ^2^), *S*	0.037, 0.125, 1.00	0.041, 0.105, 1.07	0.035, 0.111, 1.02
No. of reflections	2900	3122	3434
No. of parameters	199	217	235
H-atom treatment	H-atom parameters constrained	H-atom parameters constrained	H-atom parameters constrained
Δρ_max_, Δρ_min_ (e Å^−3^)	0.30, −0.46	0.29, −0.32	0.25, −0.60

Computer programs: *APEX2* and *SAINT* (Bruker, 2004 ▸), *SHELXS97* (Sheldrick, 2008 ▸), *SHELXL97* (Sheldrick, 2008 ▸), *ORTEP-3* for Windows (Farrugia, 2012 ▸), *PLATON* (Spek, 2009 ▸) and *publCIF* (Westrip, 2010 ▸).

## supplementary crystallographic information

### (E)-1-[4-(4-Bromobutoxy)phenyl]-3-phenylprop-2-en-1-one (I) Crystal data

**Table:**

C_19_H_19_BrO_2_	*F*(000) = 736
*M**_r_* = 359.25	*D*_x_ = 1.436 Mg m^−^^3^
Monoclinic, *P*2_1_/*c*	Mo *K*α radiation, λ = 0.71073 Å
*a* = 5.8266 (6) Å	Cell parameters from 6044 reflections
*b* = 38.743 (4) Å	θ = 2.8–21.7°
*c* = 7.5613 (7) Å	µ = 2.48 mm^−^^1^
β = 103.257 (3)°	*T* = 296 K
*V* = 1661.4 (3) Å^3^	Needle, gold
*Z* = 4	0.35 × 0.30 × 0.25 mm

### (E)-1-[4-(4-Bromobutoxy)phenyl]-3-phenylprop-2-en-1-one (I) Data collection

**Table:**

Bruker Kappa APEXII CCD diffractometer	2144 reflections with *I* > 2σ(*I*)
Bruker axs kappa axes2 CCD scans	*R*_int_ = 0.039
Absorption correction: multi-scan (SADABS; Bruker, 2004)	θ_max_ = 25.0°, θ_min_ = 2.1°
*T*_min_ = 0.485, *T*_max_ = 0.746	*h* = −6→6
23972 measured reflections	*k* = −46→46
2900 independent reflections	*l* = −8→8

### (E)-1-[4-(4-Bromobutoxy)phenyl]-3-phenylprop-2-en-1-one (I) Refinement

**Table:**

Refinement on *F*^2^	0 restraints
Least-squares matrix: full	Hydrogen site location: inferred from neighbouring sites
*R*[*F*^2^ > 2σ(*F*^2^)] = 0.037	H-atom parameters constrained
*wR*(*F*^2^) = 0.125	*w* = 1/[σ^2^(*F*_o_^2^) + (0.0813*P*)^2^ + 0.1673*P*] where *P* = (*F*_o_^2^ + 2*F*_c_^2^)/3
*S* = 1.00	(Δ/σ)_max_ < 0.001
2900 reflections	Δρ_max_ = 0.30 e Å^−^^3^
199 parameters	Δρ_min_ = −0.46 e Å^−^^3^

### (E)-1-[4-(4-Bromobutoxy)phenyl]-3-phenylprop-2-en-1-one (I) Special details

**Table:**

Geometry. All esds (except the esd in the dihedral angle between two l.s. planes) are estimated using the full covariance matrix. The cell esds are taken into account individually in the estimation of esds in distances, angles and torsion angles; correlations between esds in cell parameters are only used when they are defined by crystal symmetry. An approximate (isotropic) treatment of cell esds is used for estimating esds involving l.s. planes.

### (E)-1-[4-(4-Bromobutoxy)phenyl]-3-phenylprop-2-en-1-one (I) Fractional atomic coordinates and isotropic or equivalent isotropic displacement parameters (Å^2^)

**Table:**

	*x*	*y*	*z*	*U*_iso_*/*U*_eq_	
Br1	0.63319 (7)	0.02039 (2)	0.28807 (6)	0.0939 (2)	
O1	1.3193 (3)	0.15598 (5)	0.4937 (2)	0.0578 (5)	
O2	1.6704 (3)	0.30949 (6)	0.5960 (3)	0.0744 (6)	
C13	1.3051 (4)	0.35758 (7)	0.5004 (3)	0.0493 (6)	
H13	1.457574	0.364911	0.552739	0.059*	
C14	1.1307 (4)	0.38479 (6)	0.4445 (3)	0.0444 (6)	
C9	1.5977 (4)	0.23866 (7)	0.5833 (4)	0.0539 (7)	
H9	1.749154	0.246902	0.631214	0.065*	
C12	1.2744 (5)	0.32437 (7)	0.4863 (4)	0.0546 (7)	
H12	1.123062	0.316027	0.439286	0.066*	
C11	1.4674 (4)	0.29937 (7)	0.5411 (3)	0.0496 (6)	
C3	1.1193 (5)	0.10260 (7)	0.4330 (3)	0.0520 (7)	
H3A	1.175461	0.095894	0.559141	0.062*	
H3B	1.237229	0.095679	0.367946	0.062*	
C10	1.5605 (5)	0.20382 (7)	0.5711 (4)	0.0578 (7)	
H10	1.686048	0.188748	0.610467	0.069*	
C8	1.4155 (4)	0.26206 (7)	0.5261 (3)	0.0439 (6)	
C6	1.1532 (4)	0.21352 (7)	0.4416 (4)	0.0548 (7)	
H6	1.002310	0.205235	0.392078	0.066*	
C4	1.0918 (5)	0.14099 (7)	0.4224 (3)	0.0507 (6)	
H4A	1.032283	0.148097	0.297230	0.061*	
H4B	0.981320	0.148492	0.492860	0.061*	
C5	1.3371 (4)	0.19087 (7)	0.5003 (3)	0.0464 (6)	
C1	0.9244 (5)	0.04563 (7)	0.3742 (4)	0.0587 (7)	
H1A	0.987509	0.040162	0.501287	0.070*	
H1B	1.038552	0.038203	0.306780	0.070*	
C2	0.8918 (5)	0.08388 (6)	0.3542 (3)	0.0502 (6)	
H2A	0.771652	0.091292	0.416000	0.060*	
H2B	0.838630	0.089656	0.226542	0.060*	
C19	1.1987 (5)	0.41926 (7)	0.4658 (4)	0.0573 (7)	
H19	1.354784	0.424683	0.518908	0.069*	
C7	1.1941 (5)	0.24836 (7)	0.4565 (4)	0.0536 (6)	
H7	1.067877	0.263371	0.418390	0.064*	
C15	0.8943 (4)	0.37777 (8)	0.3649 (3)	0.0535 (7)	
H15	0.842960	0.354994	0.350901	0.064*	
C18	1.0390 (7)	0.44548 (8)	0.4097 (4)	0.0720 (9)	
H18	1.087551	0.468362	0.425886	0.086*	
C16	0.7374 (6)	0.40399 (9)	0.3074 (4)	0.0693 (8)	
H16	0.581280	0.398904	0.252541	0.083*	
C17	0.8100 (7)	0.43798 (9)	0.3303 (4)	0.0759 (10)	
H17	0.702642	0.455717	0.291748	0.091*	

### (E)-1-[4-(4-Bromobutoxy)phenyl]-3-phenylprop-2-en-1-one (I) Atomic displacement parameters (Å^2^)

**Table:**

	*U*^11^	*U*^22^	*U*^33^	*U*^12^	*U*^13^	*U*^23^
Br1	0.0793 (3)	0.0526 (3)	0.1425 (4)	−0.01202 (17)	0.0105 (3)	0.00946 (19)
O1	0.0514 (11)	0.0425 (11)	0.0732 (12)	0.0031 (9)	0.0014 (9)	0.0009 (9)
O2	0.0448 (11)	0.0562 (13)	0.1106 (16)	−0.0067 (10)	−0.0061 (11)	0.0055 (11)
C13	0.0426 (14)	0.0507 (17)	0.0530 (15)	−0.0049 (12)	0.0077 (11)	−0.0015 (12)
C14	0.0486 (14)	0.0432 (14)	0.0439 (13)	−0.0015 (11)	0.0159 (11)	−0.0026 (11)
C9	0.0357 (14)	0.0565 (17)	0.0631 (16)	0.0006 (12)	−0.0019 (12)	0.0013 (13)
C12	0.0427 (15)	0.0470 (17)	0.0689 (17)	−0.0027 (12)	0.0017 (12)	−0.0008 (13)
C11	0.0403 (14)	0.0525 (16)	0.0526 (14)	−0.0029 (12)	0.0036 (12)	0.0026 (12)
C3	0.0592 (17)	0.0469 (15)	0.0478 (14)	0.0034 (12)	0.0078 (12)	−0.0001 (12)
C10	0.0417 (15)	0.0525 (17)	0.0742 (18)	0.0114 (13)	0.0032 (13)	0.0065 (14)
C8	0.0378 (13)	0.0489 (15)	0.0436 (13)	0.0012 (11)	0.0065 (10)	0.0029 (11)
C6	0.0377 (14)	0.0500 (16)	0.0700 (17)	−0.0036 (12)	−0.0012 (12)	0.0013 (13)
C4	0.0505 (15)	0.0475 (15)	0.0529 (14)	0.0008 (12)	0.0095 (12)	−0.0012 (12)
C5	0.0476 (15)	0.0430 (15)	0.0472 (14)	0.0017 (11)	0.0079 (11)	0.0023 (11)
C1	0.0672 (18)	0.0437 (15)	0.0617 (16)	0.0014 (13)	0.0077 (13)	0.0006 (13)
C2	0.0577 (16)	0.0429 (15)	0.0488 (14)	0.0028 (12)	0.0095 (12)	0.0015 (11)
C19	0.0635 (17)	0.0482 (16)	0.0639 (16)	−0.0033 (14)	0.0223 (14)	−0.0028 (13)
C7	0.0388 (14)	0.0465 (15)	0.0699 (16)	0.0049 (12)	0.0009 (12)	0.0057 (13)
C15	0.0492 (16)	0.0563 (17)	0.0542 (15)	0.0000 (13)	0.0101 (12)	−0.0039 (12)
C18	0.096 (3)	0.0493 (18)	0.081 (2)	0.0084 (17)	0.0413 (19)	0.0064 (15)
C16	0.0599 (18)	0.086 (3)	0.0608 (17)	0.0186 (18)	0.0116 (14)	0.0076 (16)
C17	0.090 (3)	0.075 (2)	0.069 (2)	0.036 (2)	0.0307 (19)	0.0242 (17)

### (E)-1-[4-(4-Bromobutoxy)phenyl]-3-phenylprop-2-en-1-one (I) Geometric parameters (Å, º)

**Table:**

Br1—C1	1.937 (3)	C8—C7	1.383 (3)
O1—C5	1.356 (3)	C6—C7	1.371 (4)
O1—C4	1.434 (3)	C6—C5	1.377 (4)
O2—C11	1.225 (3)	C6—H6	0.9300
C13—C12	1.300 (4)	C4—H4A	0.9700
C13—C14	1.458 (4)	C4—H4B	0.9700
C13—H13	0.9300	C1—C2	1.497 (4)
C14—C19	1.392 (4)	C1—H1A	0.9700
C14—C15	1.397 (3)	C1—H1B	0.9700
C9—C10	1.367 (4)	C2—H2A	0.9700
C9—C8	1.388 (3)	C2—H2B	0.9700
C9—H9	0.9300	C19—C18	1.377 (4)
C12—C11	1.470 (4)	C19—H19	0.9300
C12—H12	0.9300	C7—H7	0.9300
C11—C8	1.475 (4)	C15—C16	1.370 (4)
C3—C4	1.496 (4)	C15—H15	0.9300
C3—C2	1.509 (4)	C18—C17	1.363 (5)
C3—H3A	0.9700	C18—H18	0.9300
C3—H3B	0.9700	C16—C17	1.382 (5)
C10—C5	1.383 (4)	C16—H16	0.9300
C10—H10	0.9300	C17—H17	0.9300
			
C5—O1—C4	118.3 (2)	C3—C4—H4B	110.2
C12—C13—C14	128.2 (2)	H4A—C4—H4B	108.5
C12—C13—H13	115.9	O1—C5—C6	125.2 (2)
C14—C13—H13	115.9	O1—C5—C10	115.7 (2)
C19—C14—C15	117.6 (3)	C6—C5—C10	119.2 (2)
C19—C14—C13	119.9 (2)	C2—C1—Br1	112.62 (19)
C15—C14—C13	122.5 (2)	C2—C1—H1A	109.1
C10—C9—C8	121.7 (2)	Br1—C1—H1A	109.1
C10—C9—H9	119.1	C2—C1—H1B	109.1
C8—C9—H9	119.1	Br1—C1—H1B	109.1
C13—C12—C11	123.2 (2)	H1A—C1—H1B	107.8
C13—C12—H12	118.4	C1—C2—C3	110.9 (2)
C11—C12—H12	118.4	C1—C2—H2A	109.5
O2—C11—C12	120.1 (2)	C3—C2—H2A	109.5
O2—C11—C8	120.3 (2)	C1—C2—H2B	109.5
C12—C11—C8	119.6 (2)	C3—C2—H2B	109.5
C4—C3—C2	112.5 (2)	H2A—C2—H2B	108.1
C4—C3—H3A	109.1	C18—C19—C14	121.2 (3)
C2—C3—H3A	109.1	C18—C19—H19	119.4
C4—C3—H3B	109.1	C14—C19—H19	119.4
C2—C3—H3B	109.1	C6—C7—C8	122.6 (2)
H3A—C3—H3B	107.8	C6—C7—H7	118.7
C9—C10—C5	120.3 (2)	C8—C7—H7	118.7
C9—C10—H10	119.8	C16—C15—C14	120.9 (3)
C5—C10—H10	119.8	C16—C15—H15	119.6
C7—C8—C9	116.6 (2)	C14—C15—H15	119.6
C7—C8—C11	124.2 (2)	C17—C18—C19	120.2 (3)
C9—C8—C11	119.2 (2)	C17—C18—H18	119.9
C7—C6—C5	119.5 (2)	C19—C18—H18	119.9
C7—C6—H6	120.2	C15—C16—C17	120.3 (3)
C5—C6—H6	120.2	C15—C16—H16	119.9
O1—C4—C3	107.7 (2)	C17—C16—H16	119.9
O1—C4—H4A	110.2	C18—C17—C16	119.9 (3)
C3—C4—H4A	110.2	C18—C17—H17	120.0
O1—C4—H4B	110.2	C16—C17—H17	120.0
			
C12—C13—C14—C19	−178.0 (3)	C7—C6—C5—C10	−0.9 (4)
C12—C13—C14—C15	0.4 (4)	C9—C10—C5—O1	179.8 (2)
C14—C13—C12—C11	177.4 (2)	C9—C10—C5—C6	0.4 (4)
C13—C12—C11—O2	−3.2 (4)	Br1—C1—C2—C3	176.61 (18)
C13—C12—C11—C8	178.0 (3)	C4—C3—C2—C1	−177.7 (2)
C8—C9—C10—C5	0.0 (4)	C15—C14—C19—C18	−0.3 (4)
C10—C9—C8—C7	0.2 (4)	C13—C14—C19—C18	178.2 (2)
C10—C9—C8—C11	−179.4 (2)	C5—C6—C7—C8	1.1 (4)
O2—C11—C8—C7	−176.4 (3)	C9—C8—C7—C6	−0.8 (4)
C12—C11—C8—C7	2.4 (4)	C11—C8—C7—C6	178.7 (2)
O2—C11—C8—C9	3.1 (4)	C19—C14—C15—C16	1.2 (4)
C12—C11—C8—C9	−178.1 (2)	C13—C14—C15—C16	−177.3 (2)
C5—O1—C4—C3	−179.0 (2)	C14—C19—C18—C17	−0.5 (4)
C2—C3—C4—O1	−177.7 (2)	C14—C15—C16—C17	−1.2 (4)
C4—O1—C5—C6	−0.9 (4)	C19—C18—C17—C16	0.5 (5)
C4—O1—C5—C10	179.7 (2)	C15—C16—C17—C18	0.4 (5)
C7—C6—C5—O1	179.7 (2)		

### (E)-1-[4-(4-Bromobutoxy)phenyl]-3-phenylprop-2-en-1-one (I) Hydrogen-bond geometry (Å, º)

Cg is the centroid of the C14–C19 ring

**Table:**

*D*—H···*A*	*D*—H	H···*A*	*D*···*A*	*D*—H···*A*
C2—H2*B*···*Cg*^i^	0.97	2.84	3.664 (3)	144

Symmetry code: (i) *x*, −*y*−1/2, *z*−3/2.

### (E)-1-[4-(4-Bromobutoxy)phenyl]-3-(4-methoxyphenyl)prop-2-en-1-one (II) Crystal data

**Table:**

C_20_H_21_BrO_3_	*F*(000) = 800
*M**_r_* = 389.28	*D*_x_ = 1.443 Mg m^−^^3^
Monoclinic, *P*2_1_/*c*	Mo *K*α radiation, λ = 0.71073 Å
*a* = 5.7331 (3) Å	Cell parameters from 7354 reflections
*b* = 41.732 (2) Å	θ = 2.8–22.9°
*c* = 7.6476 (4) Å	µ = 2.31 mm^−^^1^
β = 101.767 (2)°	*T* = 296 K
*V* = 1791.28 (16) Å^3^	Block, yellow
*Z* = 4	0.35 × 0.30 × 0.25 mm

### (E)-1-[4-(4-Bromobutoxy)phenyl]-3-(4-methoxyphenyl)prop-2-en-1-one (II) Data collection

**Table:**

Bruker Kappa APEXII CCD diffractometer	2467 reflections with *I* > 2σ(*I*)
Bruker axs kappa axes2 CCD scans	*R*_int_ = 0.028
Absorption correction: multi-scan (SADABS; Bruker, 2004)	θ_max_ = 25.0°, θ_min_ = 2.8°
*T*_min_ = 0.639, *T*_max_ = 0.746	*h* = −6→6
21484 measured reflections	*k* = −49→48
3122 independent reflections	*l* = −9→9

### (E)-1-[4-(4-Bromobutoxy)phenyl]-3-(4-methoxyphenyl)prop-2-en-1-one (II) Refinement

**Table:**

Refinement on *F*^2^	0 restraints
Least-squares matrix: full	Hydrogen site location: inferred from neighbouring sites
*R*[*F*^2^ > 2σ(*F*^2^)] = 0.041	H-atom parameters constrained
*wR*(*F*^2^) = 0.105	*w* = 1/[σ^2^(*F*_o_^2^) + (0.0437*P*)^2^ + 1.2667*P*] where *P* = (*F*_o_^2^ + 2*F*_c_^2^)/3
*S* = 1.07	(Δ/σ)_max_ < 0.001
3122 reflections	Δρ_max_ = 0.29 e Å^−^^3^
217 parameters	Δρ_min_ = −0.32 e Å^−^^3^

### (E)-1-[4-(4-Bromobutoxy)phenyl]-3-(4-methoxyphenyl)prop-2-en-1-one (II) Special details

**Table:**

Geometry. All esds (except the esd in the dihedral angle between two l.s. planes) are estimated using the full covariance matrix. The cell esds are taken into account individually in the estimation of esds in distances, angles and torsion angles; correlations between esds in cell parameters are only used when they are defined by crystal symmetry. An approximate (isotropic) treatment of cell esds is used for estimating esds involving l.s. planes.

### (E)-1-[4-(4-Bromobutoxy)phenyl]-3-(4-methoxyphenyl)prop-2-en-1-one (II) Fractional atomic coordinates and isotropic or equivalent isotropic displacement parameters (Å^2^)

**Table:**

	*x*	*y*	*z*	*U*_iso_*/*U*_eq_	
Br1	0.80559 (7)	0.03512 (2)	0.33626 (5)	0.07602 (19)	
O1	0.1223 (4)	0.16146 (5)	0.1086 (3)	0.0566 (6)	
O2	−0.2411 (4)	0.30384 (5)	0.0038 (4)	0.0701 (7)	
C19	0.5345 (5)	0.36791 (7)	0.2387 (4)	0.0463 (7)	
H19	0.586875	0.346778	0.249645	0.056*	
C14	0.2970 (5)	0.37443 (7)	0.1604 (4)	0.0402 (6)	
C12	0.1574 (5)	0.31803 (7)	0.1132 (4)	0.0509 (8)	
H12	0.310553	0.310405	0.157192	0.061*	
C8	0.0189 (5)	0.25991 (6)	0.0735 (4)	0.0413 (7)	
C17	0.6188 (6)	0.42374 (7)	0.2804 (4)	0.0502 (8)	
O3	0.7918 (4)	0.44592 (6)	0.3447 (3)	0.0728 (7)	
C15	0.2296 (5)	0.40641 (7)	0.1432 (4)	0.0488 (7)	
H15	0.073085	0.411435	0.090331	0.059*	
C5	0.1019 (5)	0.19408 (7)	0.1012 (4)	0.0450 (7)	
C13	0.1236 (5)	0.34903 (7)	0.1027 (4)	0.0447 (7)	
H13	−0.030148	0.355709	0.052036	0.054*	
C10	0.2842 (5)	0.21506 (7)	0.1628 (4)	0.0531 (8)	
H10	0.434922	0.207490	0.215004	0.064*	
C18	0.6913 (5)	0.39211 (8)	0.2995 (4)	0.0517 (8)	
H18	0.847485	0.387247	0.353949	0.062*	
C4	0.3497 (5)	0.14792 (7)	0.1833 (4)	0.0518 (8)	
H4A	0.401764	0.154805	0.306220	0.062*	
H4B	0.467011	0.154770	0.115952	0.062*	
C1	0.5163 (6)	0.05930 (7)	0.2405 (5)	0.0580 (8)	
H1A	0.461700	0.053788	0.115649	0.070*	
H1B	0.392821	0.053086	0.303685	0.070*	
C11	−0.0366 (5)	0.29464 (7)	0.0585 (4)	0.0478 (7)	
C16	0.3876 (6)	0.43108 (7)	0.2019 (4)	0.0533 (8)	
H16	0.337947	0.452317	0.188410	0.064*	
C2	0.5516 (5)	0.09482 (7)	0.2563 (4)	0.0499 (7)	
H2A	0.677114	0.101216	0.195511	0.060*	
H2B	0.600138	0.100671	0.381178	0.060*	
C7	−0.1621 (5)	0.23800 (7)	0.0135 (4)	0.0529 (8)	
H7	−0.313953	0.245422	−0.036794	0.063*	
C6	−0.1214 (5)	0.20577 (7)	0.0269 (4)	0.0554 (8)	
H6	−0.245411	0.191563	−0.014460	0.066*	
C9	0.2409 (5)	0.24765 (7)	0.1464 (5)	0.0534 (8)	
H9	0.365956	0.261794	0.185820	0.064*	
C3	0.3229 (6)	0.11218 (7)	0.1747 (4)	0.0509 (7)	
H3A	0.273050	0.105718	0.050870	0.061*	
H3B	0.198973	0.105884	0.237200	0.061*	
C20	0.7321 (8)	0.47862 (9)	0.3209 (6)	0.0839 (12)	
H20A	0.867967	0.491478	0.371571	0.126*	
H20B	0.684671	0.483197	0.195622	0.126*	
H20C	0.603081	0.483462	0.379191	0.126*	

### (E)-1-[4-(4-Bromobutoxy)phenyl]-3-(4-methoxyphenyl)prop-2-en-1-one (II) Atomic displacement parameters (Å^2^)

**Table:**

	*U*^11^	*U*^22^	*U*^33^	*U*^12^	*U*^13^	*U*^23^
Br1	0.0824 (3)	0.0585 (3)	0.0824 (3)	0.01893 (19)	0.0055 (2)	−0.00496 (19)
O1	0.0525 (12)	0.0385 (12)	0.0737 (15)	−0.0032 (10)	0.0011 (11)	−0.0014 (10)
O2	0.0411 (13)	0.0529 (14)	0.107 (2)	0.0063 (11)	−0.0062 (12)	−0.0058 (13)
C19	0.0432 (17)	0.0411 (17)	0.0541 (18)	0.0060 (13)	0.0093 (14)	0.0019 (14)
C14	0.0422 (16)	0.0396 (16)	0.0406 (16)	0.0012 (13)	0.0126 (13)	0.0028 (12)
C12	0.0398 (16)	0.0430 (18)	0.066 (2)	0.0016 (13)	0.0009 (15)	0.0016 (14)
C8	0.0361 (15)	0.0417 (16)	0.0448 (16)	−0.0009 (12)	0.0053 (13)	−0.0031 (13)
C17	0.057 (2)	0.0503 (19)	0.0467 (17)	−0.0120 (15)	0.0172 (15)	−0.0077 (14)
O3	0.0712 (16)	0.0618 (16)	0.0834 (17)	−0.0202 (12)	0.0109 (13)	−0.0152 (13)
C15	0.0441 (17)	0.0441 (17)	0.0583 (19)	0.0049 (14)	0.0109 (14)	0.0065 (14)
C5	0.0471 (17)	0.0377 (16)	0.0490 (17)	−0.0012 (13)	0.0074 (14)	−0.0025 (13)
C13	0.0388 (15)	0.0430 (17)	0.0512 (18)	0.0067 (13)	0.0067 (13)	0.0019 (13)
C10	0.0372 (16)	0.0435 (18)	0.073 (2)	0.0032 (14)	−0.0013 (15)	−0.0033 (15)
C18	0.0434 (17)	0.060 (2)	0.0498 (18)	0.0000 (15)	0.0051 (14)	0.0019 (15)
C4	0.0533 (19)	0.0438 (17)	0.0581 (19)	0.0022 (14)	0.0110 (15)	−0.0009 (15)
C1	0.068 (2)	0.0471 (18)	0.058 (2)	0.0068 (16)	0.0088 (16)	−0.0042 (15)
C11	0.0407 (17)	0.0447 (17)	0.0559 (18)	0.0025 (13)	0.0048 (14)	−0.0024 (14)
C16	0.062 (2)	0.0389 (17)	0.062 (2)	0.0008 (15)	0.0195 (17)	0.0012 (14)
C2	0.0577 (19)	0.0441 (18)	0.0477 (18)	0.0000 (14)	0.0103 (15)	−0.0031 (14)
C7	0.0349 (16)	0.0516 (19)	0.066 (2)	−0.0001 (14)	−0.0037 (15)	−0.0001 (15)
C6	0.0423 (17)	0.0443 (18)	0.074 (2)	−0.0101 (14)	−0.0015 (16)	−0.0054 (16)
C9	0.0394 (17)	0.0438 (17)	0.072 (2)	−0.0061 (13)	0.0001 (15)	−0.0069 (15)
C3	0.0592 (19)	0.0428 (17)	0.0511 (18)	−0.0018 (14)	0.0119 (15)	−0.0028 (14)
C20	0.110 (3)	0.060 (2)	0.085 (3)	−0.035 (2)	0.028 (2)	−0.015 (2)

### (E)-1-[4-(4-Bromobutoxy)phenyl]-3-(4-methoxyphenyl)prop-2-en-1-one (II) Geometric parameters (Å, º)

**Table:**

Br1—C1	1.952 (3)	C13—H13	0.9300
O1—C5	1.367 (3)	C10—C9	1.384 (4)
O1—C4	1.429 (4)	C10—H10	0.9300
O2—C11	1.223 (3)	C18—H18	0.9300
C19—C18	1.369 (4)	C4—C3	1.499 (4)
C19—C14	1.398 (4)	C4—H4A	0.9700
C19—H19	0.9300	C4—H4B	0.9700
C14—C15	1.388 (4)	C1—C2	1.498 (4)
C14—C13	1.459 (4)	C1—H1A	0.9700
C12—C13	1.308 (4)	C1—H1B	0.9700
C12—C11	1.475 (4)	C16—H16	0.9300
C12—H12	0.9300	C2—C3	1.517 (4)
C8—C9	1.380 (4)	C2—H2A	0.9700
C8—C7	1.389 (4)	C2—H2B	0.9700
C8—C11	1.483 (4)	C7—C6	1.365 (4)
C17—O3	1.372 (4)	C7—H7	0.9300
C17—C16	1.373 (4)	C6—H6	0.9300
C17—C18	1.383 (4)	C9—H9	0.9300
O3—C20	1.410 (5)	C3—H3A	0.9700
C15—C16	1.384 (4)	C3—H3B	0.9700
C15—H15	0.9300	C20—H20A	0.9600
C5—C10	1.372 (4)	C20—H20B	0.9600
C5—C6	1.380 (4)	C20—H20C	0.9600
			
C5—O1—C4	118.3 (2)	C2—C1—H1A	109.0
C18—C19—C14	121.1 (3)	Br1—C1—H1A	109.0
C18—C19—H19	119.4	C2—C1—H1B	109.0
C14—C19—H19	119.4	Br1—C1—H1B	109.0
C15—C14—C19	117.1 (3)	H1A—C1—H1B	107.8
C15—C14—C13	120.7 (3)	O2—C11—C12	120.3 (3)
C19—C14—C13	122.2 (3)	O2—C11—C8	120.6 (3)
C13—C12—C11	122.9 (3)	C12—C11—C8	119.2 (3)
C13—C12—H12	118.5	C17—C16—C15	119.0 (3)
C11—C12—H12	118.5	C17—C16—H16	120.5
C9—C8—C7	117.1 (3)	C15—C16—H16	120.5
C9—C8—C11	124.0 (3)	C1—C2—C3	110.4 (3)
C7—C8—C11	118.9 (3)	C1—C2—H2A	109.6
O3—C17—C16	124.7 (3)	C3—C2—H2A	109.6
O3—C17—C18	115.2 (3)	C1—C2—H2B	109.6
C16—C17—C18	120.1 (3)	C3—C2—H2B	109.6
C17—O3—C20	117.9 (3)	H2A—C2—H2B	108.1
C16—C15—C14	122.2 (3)	C6—C7—C8	121.3 (3)
C16—C15—H15	118.9	C6—C7—H7	119.4
C14—C15—H15	118.9	C8—C7—H7	119.4
O1—C5—C10	124.7 (3)	C7—C6—C5	120.6 (3)
O1—C5—C6	115.7 (3)	C7—C6—H6	119.7
C10—C5—C6	119.6 (3)	C5—C6—H6	119.7
C12—C13—C14	128.1 (3)	C8—C9—C10	122.3 (3)
C12—C13—H13	116.0	C8—C9—H9	118.8
C14—C13—H13	116.0	C10—C9—H9	118.8
C5—C10—C9	119.1 (3)	C4—C3—C2	112.6 (3)
C5—C10—H10	120.4	C4—C3—H3A	109.1
C9—C10—H10	120.4	C2—C3—H3A	109.1
C19—C18—C17	120.4 (3)	C4—C3—H3B	109.1
C19—C18—H18	119.8	C2—C3—H3B	109.1
C17—C18—H18	119.8	H3A—C3—H3B	107.8
O1—C4—C3	107.4 (2)	O3—C20—H20A	109.5
O1—C4—H4A	110.2	O3—C20—H20B	109.5
C3—C4—H4A	110.2	H20A—C20—H20B	109.5
O1—C4—H4B	110.2	O3—C20—H20C	109.5
C3—C4—H4B	110.2	H20A—C20—H20C	109.5
H4A—C4—H4B	108.5	H20B—C20—H20C	109.5
C2—C1—Br1	113.0 (2)		
			
C18—C19—C14—C15	1.6 (4)	C9—C8—C11—O2	−174.9 (3)
C18—C19—C14—C13	−176.9 (3)	C7—C8—C11—O2	4.1 (5)
C16—C17—O3—C20	−3.3 (4)	C9—C8—C11—C12	4.1 (5)
C18—C17—O3—C20	176.8 (3)	C7—C8—C11—C12	−176.8 (3)
C19—C14—C15—C16	−0.7 (4)	O3—C17—C16—C15	−179.7 (3)
C13—C14—C15—C16	177.8 (3)	C18—C17—C16—C15	0.2 (4)
C4—O1—C5—C10	−0.9 (4)	C14—C15—C16—C17	−0.2 (5)
C4—O1—C5—C6	179.7 (3)	Br1—C1—C2—C3	178.3 (2)
C11—C12—C13—C14	176.6 (3)	C9—C8—C7—C6	0.2 (5)
C15—C14—C13—C12	−178.5 (3)	C11—C8—C7—C6	−178.9 (3)
C19—C14—C13—C12	0.0 (5)	C8—C7—C6—C5	0.1 (5)
O1—C5—C10—C9	179.5 (3)	O1—C5—C6—C7	179.8 (3)
C6—C5—C10—C9	−1.1 (5)	C10—C5—C6—C7	0.3 (5)
C14—C19—C18—C17	−1.7 (4)	C7—C8—C9—C10	−1.0 (5)
O3—C17—C18—C19	−179.4 (3)	C11—C8—C9—C10	178.0 (3)
C16—C17—C18—C19	0.8 (4)	C5—C10—C9—C8	1.5 (5)
C5—O1—C4—C3	−179.8 (2)	O1—C4—C3—C2	−178.1 (2)
C13—C12—C11—O2	−1.6 (5)	C1—C2—C3—C4	−178.8 (3)
C13—C12—C11—C8	179.4 (3)		

### (E)-1-[4-(4-Bromobutoxy)phenyl]-3-(4-methoxyphenyl)prop-2-en-1-one (II) Hydrogen-bond geometry (Å, º)

Cg is the centroid of the C14–C19 ring.

**Table:**

*D*—H···*A*	*D*—H	H···*A*	*D*···*A*	*D*—H···*A*
C2—H2*B*···*Cg*^i^	0.97	2.87	3.703 (3)	144
C3—H3*A*···*Cg*^ii^	0.97	2.94	3.743 (3)	140

Symmetry codes: (i) *x*, −*y*−1/2, *z*−1/2; (ii) *x*, −*y*−1/2, *z*−3/2.

### (E)-1-[4-(4-Bromobutoxy)phenyl]-3-(3,4-dimethoxyphenyl)prop-2-en-1-one (III) Crystal data

**Table:**

C_21_H_23_BrO_4_	*F*(000) = 864
*M**_r_* = 419.30	*D*_x_ = 1.432 Mg m^−^^3^
Monoclinic, *P*2_1_/*c*	Mo *K*α radiation, λ = 0.71073 Å
*a* = 9.4765 (4) Å	Cell parameters from 6542 reflections
*b* = 26.0984 (12) Å	θ = 2.3–22.8°
*c* = 7.8666 (4) Å	µ = 2.14 mm^−^^1^
β = 91.427 (2)°	*T* = 296 K
*V* = 1944.98 (16) Å^3^	Block, yellow
*Z* = 4	0.35 × 0.30 × 0.25 mm

### (E)-1-[4-(4-Bromobutoxy)phenyl]-3-(3,4-dimethoxyphenyl)prop-2-en-1-one (III) Data collection

**Table:**

Bruker Kappa APEXII CCD diffractometer	2416 reflections with *I* > 2σ(*I*)
Bruker axs kappa axes2 CCD scans	*R*_int_ = 0.043
Absorption correction: multi-scan (SADABS; Bruker, 2004)	θ_max_ = 25.0°, θ_min_ = 2.2°
*T*_min_ = 0.667, *T*_max_ = 0.746	*h* = −10→11
28826 measured reflections	*k* = −31→31
3434 independent reflections	*l* = −7→9

### (E)-1-[4-(4-Bromobutoxy)phenyl]-3-(3,4-dimethoxyphenyl)prop-2-en-1-one (III) Refinement

**Table:**

Refinement on *F*^2^	0 restraints
Least-squares matrix: full	Hydrogen site location: inferred from neighbouring sites
*R*[*F*^2^ > 2σ(*F*^2^)] = 0.035	H-atom parameters constrained
*wR*(*F*^2^) = 0.111	*w* = 1/[σ^2^(*F*_o_^2^) + (0.0612*P*)^2^ + 0.5481*P*] where *P* = (*F*_o_^2^ + 2*F*_c_^2^)/3
*S* = 1.02	(Δ/σ)_max_ = 0.002
3434 reflections	Δρ_max_ = 0.25 e Å^−^^3^
235 parameters	Δρ_min_ = −0.60 e Å^−^^3^

### (E)-1-[4-(4-Bromobutoxy)phenyl]-3-(3,4-dimethoxyphenyl)prop-2-en-1-one (III) Special details

**Table:**

Geometry. All esds (except the esd in the dihedral angle between two l.s. planes) are estimated using the full covariance matrix. The cell esds are taken into account individually in the estimation of esds in distances, angles and torsion angles; correlations between esds in cell parameters are only used when they are defined by crystal symmetry. An approximate (isotropic) treatment of cell esds is used for estimating esds involving l.s. planes.

### (E)-1-[4-(4-Bromobutoxy)phenyl]-3-(3,4-dimethoxyphenyl)prop-2-en-1-one (III) Fractional atomic coordinates and isotropic or equivalent isotropic displacement parameters (Å^2^)

**Table:**

	*x*	*y*	*z*	*U*_iso_*/*U*_eq_	
Br1	0.45552 (4)	0.54423 (2)	0.24312 (5)	0.07597 (19)	
O2	−0.0404 (2)	0.94255 (7)	0.1625 (3)	0.0511 (5)	
O1	0.2713 (2)	0.73775 (7)	0.3764 (3)	0.0588 (6)	
C12	0.1316 (3)	0.98125 (11)	0.3389 (3)	0.0437 (7)	
H12	0.184058	0.977372	0.439618	0.052*	
O3	0.3615 (2)	1.21305 (7)	0.4837 (3)	0.0574 (6)	
O4	0.3614 (2)	1.13839 (7)	0.6921 (2)	0.0502 (5)	
C18	0.3015 (3)	1.12792 (10)	0.5363 (3)	0.0373 (6)	
C17	0.3004 (3)	1.16949 (10)	0.4216 (3)	0.0403 (7)	
C19	0.2448 (3)	1.08188 (10)	0.4870 (3)	0.0363 (6)	
H19	0.246728	1.054409	0.562376	0.044*	
C11	0.0599 (3)	0.93675 (11)	0.2626 (3)	0.0390 (6)	
C8	0.1125 (3)	0.88440 (10)	0.3020 (3)	0.0381 (6)	
C13	0.1224 (3)	1.02697 (10)	0.2659 (3)	0.0397 (6)	
H13	0.070053	1.028283	0.164415	0.048*	
C7	0.0262 (3)	0.84234 (11)	0.2685 (4)	0.0462 (7)	
H7	−0.065785	0.847643	0.228455	0.055*	
C5	0.2117 (3)	0.78456 (11)	0.3500 (4)	0.0454 (7)	
C14	0.1837 (3)	1.07568 (10)	0.3236 (3)	0.0381 (6)	
C4	0.1895 (3)	0.69302 (10)	0.3374 (4)	0.0482 (7)	
H4A	0.158524	0.693377	0.218959	0.058*	
H4B	0.106919	0.691679	0.407688	0.058*	
C2	0.2054 (3)	0.59737 (11)	0.3392 (4)	0.0510 (7)	
H2A	0.120078	0.597264	0.404769	0.061*	
H2B	0.177261	0.596072	0.219901	0.061*	
C1	0.2885 (4)	0.54993 (11)	0.3820 (4)	0.0580 (8)	
H1A	0.229146	0.520054	0.363672	0.070*	
H1B	0.317167	0.550754	0.501159	0.070*	
C15	0.1820 (3)	1.11719 (11)	0.2142 (3)	0.0450 (7)	
H15	0.140748	1.113760	0.106332	0.054*	
C9	0.2493 (3)	0.87505 (11)	0.3629 (4)	0.0463 (7)	
H9	0.308367	0.902573	0.388830	0.056*	
C6	0.0741 (3)	0.79281 (11)	0.2934 (4)	0.0495 (7)	
H6	0.014240	0.765189	0.272127	0.059*	
C3	0.2834 (3)	0.64750 (10)	0.3727 (4)	0.0490 (7)	
H3A	0.316565	0.648451	0.490286	0.059*	
H3B	0.365060	0.649274	0.301001	0.059*	
C16	0.2404 (3)	1.16361 (11)	0.2622 (4)	0.0459 (7)	
H16	0.239027	1.190946	0.186297	0.055*	
C21	0.3812 (3)	1.09715 (11)	0.8078 (3)	0.0524 (8)	
H21A	0.423884	1.109689	0.911655	0.079*	
H21B	0.291523	1.081947	0.831299	0.079*	
H21C	0.441599	1.071909	0.758732	0.079*	
C10	0.2989 (3)	0.82590 (11)	0.3855 (4)	0.0491 (7)	
H10	0.391047	0.820470	0.424669	0.059*	
C20	0.3838 (5)	1.25427 (12)	0.3693 (5)	0.0744 (11)	
H20A	0.427353	1.282338	0.429540	0.112*	
H20B	0.444373	1.243150	0.280416	0.112*	
H20C	0.294923	1.265157	0.320543	0.112*	

### (E)-1-[4-(4-Bromobutoxy)phenyl]-3-(3,4-dimethoxyphenyl)prop-2-en-1-one (III) Atomic displacement parameters (Å^2^)

**Table:**

	*U*^11^	*U*^22^	*U*^33^	*U*^12^	*U*^13^	*U*^23^
Br1	0.0631 (3)	0.0526 (2)	0.1133 (4)	0.00316 (16)	0.0227 (2)	−0.00490 (19)
O2	0.0505 (13)	0.0438 (11)	0.0583 (12)	−0.0026 (10)	−0.0138 (11)	−0.0025 (10)
O1	0.0550 (13)	0.0367 (11)	0.0842 (16)	0.0023 (10)	−0.0073 (12)	0.0014 (10)
C12	0.0472 (17)	0.0408 (17)	0.0427 (16)	−0.0005 (14)	−0.0062 (13)	−0.0056 (13)
O3	0.0856 (16)	0.0348 (11)	0.0510 (12)	−0.0151 (11)	−0.0122 (11)	0.0071 (9)
O4	0.0747 (14)	0.0341 (10)	0.0409 (11)	−0.0102 (10)	−0.0140 (10)	0.0021 (9)
C18	0.0406 (15)	0.0343 (15)	0.0371 (15)	0.0008 (12)	0.0009 (12)	−0.0011 (12)
C17	0.0479 (16)	0.0282 (14)	0.0446 (17)	−0.0021 (12)	0.0006 (13)	0.0002 (12)
C19	0.0408 (15)	0.0293 (13)	0.0389 (15)	0.0009 (11)	0.0007 (12)	0.0014 (11)
C11	0.0403 (16)	0.0406 (15)	0.0362 (15)	−0.0012 (13)	0.0038 (13)	−0.0057 (12)
C8	0.0400 (16)	0.0379 (15)	0.0364 (14)	−0.0017 (12)	0.0023 (12)	−0.0062 (12)
C13	0.0393 (15)	0.0411 (15)	0.0387 (15)	0.0001 (13)	−0.0018 (12)	−0.0055 (13)
C7	0.0391 (16)	0.0436 (17)	0.0558 (18)	0.0001 (13)	−0.0007 (14)	−0.0053 (14)
C5	0.0507 (18)	0.0370 (16)	0.0487 (17)	−0.0008 (14)	0.0047 (14)	−0.0007 (13)
C14	0.0367 (15)	0.0353 (15)	0.0423 (16)	0.0017 (12)	0.0016 (12)	−0.0043 (12)
C4	0.0553 (18)	0.0391 (16)	0.0502 (17)	−0.0030 (14)	0.0029 (14)	−0.0028 (14)
C2	0.0493 (18)	0.0449 (17)	0.0591 (19)	−0.0034 (14)	0.0081 (15)	−0.0005 (15)
C1	0.0569 (19)	0.0420 (18)	0.075 (2)	−0.0077 (15)	0.0098 (17)	0.0010 (15)
C15	0.0504 (17)	0.0468 (17)	0.0374 (15)	0.0001 (14)	−0.0079 (13)	0.0017 (13)
C9	0.0459 (18)	0.0415 (17)	0.0514 (18)	−0.0071 (13)	0.0000 (14)	−0.0036 (14)
C6	0.0472 (18)	0.0391 (17)	0.0619 (19)	−0.0046 (13)	−0.0032 (15)	−0.0037 (14)
C3	0.0525 (18)	0.0374 (16)	0.0572 (19)	0.0002 (14)	0.0002 (15)	0.0013 (14)
C16	0.0570 (18)	0.0381 (16)	0.0425 (17)	−0.0026 (14)	−0.0034 (14)	0.0099 (13)
C21	0.065 (2)	0.0448 (17)	0.0468 (17)	−0.0041 (15)	−0.0155 (15)	0.0087 (14)
C10	0.0411 (16)	0.0454 (17)	0.0604 (19)	0.0021 (14)	−0.0046 (14)	0.0001 (14)
C20	0.112 (3)	0.0412 (18)	0.069 (2)	−0.024 (2)	−0.013 (2)	0.0165 (17)

### (E)-1-[4-(4-Bromobutoxy)phenyl]-3-(3,4-dimethoxyphenyl)prop-2-en-1-one (III) Geometric parameters (Å, º)

**Table:**

Br1—C1	1.951 (3)	C14—C15	1.383 (4)
O2—C11	1.228 (3)	C4—C3	1.506 (4)
O1—C5	1.360 (3)	C4—H4A	0.9700
O1—C4	1.430 (3)	C4—H4B	0.9700
C12—C13	1.326 (4)	C2—C1	1.501 (4)
C12—C11	1.467 (4)	C2—C3	1.522 (4)
C12—H12	0.9300	C2—H2A	0.9700
O3—C17	1.361 (3)	C2—H2B	0.9700
O3—C20	1.422 (3)	C1—H1A	0.9700
O4—C18	1.366 (3)	C1—H1B	0.9700
O4—C21	1.419 (3)	C15—C16	1.380 (4)
C18—C19	1.368 (4)	C15—H15	0.9300
C18—C17	1.411 (4)	C9—C10	1.376 (4)
C17—C16	1.373 (4)	C9—H9	0.9300
C19—C14	1.406 (4)	C6—H6	0.9300
C19—H19	0.9300	C3—H3A	0.9700
C11—C8	1.484 (4)	C3—H3B	0.9700
C8—C7	1.390 (4)	C16—H16	0.9300
C8—C9	1.393 (4)	C21—H21A	0.9600
C13—C14	1.465 (4)	C21—H21B	0.9600
C13—H13	0.9300	C21—H21C	0.9600
C7—C6	1.382 (4)	C10—H10	0.9300
C7—H7	0.9300	C20—H20A	0.9600
C5—C10	1.383 (4)	C20—H20B	0.9600
C5—C6	1.384 (4)	C20—H20C	0.9600
			
C5—O1—C4	118.7 (2)	C1—C2—H2B	108.5
C13—C12—C11	120.7 (3)	C3—C2—H2B	108.5
C13—C12—H12	119.7	H2A—C2—H2B	107.5
C11—C12—H12	119.7	C2—C1—Br1	111.5 (2)
C17—O3—C20	118.3 (2)	C2—C1—H1A	109.3
C18—O4—C21	118.0 (2)	Br1—C1—H1A	109.3
O4—C18—C19	125.5 (2)	C2—C1—H1B	109.3
O4—C18—C17	114.6 (2)	Br1—C1—H1B	109.3
C19—C18—C17	119.8 (2)	H1A—C1—H1B	108.0
O3—C17—C16	125.7 (2)	C16—C15—C14	121.3 (2)
O3—C17—C18	114.6 (2)	C16—C15—H15	119.4
C16—C17—C18	119.6 (2)	C14—C15—H15	119.4
C18—C19—C14	120.6 (2)	C10—C9—C8	121.3 (3)
C18—C19—H19	119.7	C10—C9—H9	119.4
C14—C19—H19	119.7	C8—C9—H9	119.4
O2—C11—C12	120.5 (3)	C7—C6—C5	119.6 (3)
O2—C11—C8	119.8 (2)	C7—C6—H6	120.2
C12—C11—C8	119.6 (2)	C5—C6—H6	120.2
C7—C8—C9	117.7 (3)	C4—C3—C2	111.4 (2)
C7—C8—C11	119.6 (2)	C4—C3—H3A	109.4
C9—C8—C11	122.5 (2)	C2—C3—H3A	109.4
C12—C13—C14	128.7 (3)	C4—C3—H3B	109.4
C12—C13—H13	115.7	C2—C3—H3B	109.4
C14—C13—H13	115.7	H3A—C3—H3B	108.0
C6—C7—C8	121.5 (3)	C17—C16—C15	120.1 (2)
C6—C7—H7	119.3	C17—C16—H16	119.9
C8—C7—H7	119.3	C15—C16—H16	119.9
O1—C5—C10	115.2 (3)	O4—C21—H21A	109.5
O1—C5—C6	125.0 (3)	O4—C21—H21B	109.5
C10—C5—C6	119.8 (3)	H21A—C21—H21B	109.5
C15—C14—C19	118.5 (2)	O4—C21—H21C	109.5
C15—C14—C13	119.2 (2)	H21A—C21—H21C	109.5
C19—C14—C13	122.3 (2)	H21B—C21—H21C	109.5
O1—C4—C3	106.9 (2)	C9—C10—C5	120.1 (3)
O1—C4—H4A	110.4	C9—C10—H10	120.0
C3—C4—H4A	110.4	C5—C10—H10	120.0
O1—C4—H4B	110.4	O3—C20—H20A	109.5
C3—C4—H4B	110.4	O3—C20—H20B	109.5
H4A—C4—H4B	108.6	H20A—C20—H20B	109.5
C1—C2—C3	114.9 (3)	O3—C20—H20C	109.5
C1—C2—H2A	108.5	H20A—C20—H20C	109.5
C3—C2—H2A	108.5	H20B—C20—H20C	109.5
			
C21—O4—C18—C19	7.3 (4)	C18—C19—C14—C15	−0.1 (4)
C21—O4—C18—C17	−172.9 (3)	C18—C19—C14—C13	179.7 (2)
C20—O3—C17—C16	−9.3 (5)	C12—C13—C14—C15	168.9 (3)
C20—O3—C17—C18	170.8 (3)	C12—C13—C14—C19	−11.0 (4)
O4—C18—C17—O3	1.2 (4)	C5—O1—C4—C3	177.9 (2)
C19—C18—C17—O3	−179.1 (2)	C3—C2—C1—Br1	−62.6 (3)
O4—C18—C17—C16	−178.8 (3)	C19—C14—C15—C16	0.9 (4)
C19—C18—C17—C16	1.0 (4)	C13—C14—C15—C16	−179.0 (3)
O4—C18—C19—C14	178.9 (2)	C7—C8—C9—C10	1.5 (4)
C17—C18—C19—C14	−0.8 (4)	C11—C8—C9—C10	−174.7 (3)
C13—C12—C11—O2	−21.5 (4)	C8—C7—C6—C5	−1.1 (4)
C13—C12—C11—C8	156.3 (3)	O1—C5—C6—C7	−178.9 (3)
O2—C11—C8—C7	−19.1 (4)	C10—C5—C6—C7	1.7 (4)
C12—C11—C8—C7	163.1 (2)	O1—C4—C3—C2	178.7 (2)
O2—C11—C8—C9	157.1 (3)	C1—C2—C3—C4	−176.2 (3)
C12—C11—C8—C9	−20.8 (4)	O3—C17—C16—C15	179.8 (3)
C11—C12—C13—C14	178.8 (3)	C18—C17—C16—C15	−0.3 (4)
C9—C8—C7—C6	−0.4 (4)	C14—C15—C16—C17	−0.7 (5)
C11—C8—C7—C6	175.9 (3)	C8—C9—C10—C5	−1.0 (4)
C4—O1—C5—C10	−178.0 (2)	O1—C5—C10—C9	179.9 (3)
C4—O1—C5—C6	2.5 (4)	C6—C5—C10—C9	−0.6 (4)

### (E)-1-[4-(4-Bromobutoxy)phenyl]-3-(3,4-dimethoxyphenyl)prop-2-en-1-one (III) Hydrogen-bond geometry (Å, º)

**Table:**

*D*—H···*A*	*D*—H	H···*A*	*D*···*A*	*D*—H···*A*
C10—H10···O3^i^	0.93	2.59	3.505 (3)	169

Symmetry code: (i) −*x*+1, −*y*+2, −*z*+1.
